# Game Elements in the Design of Simulations in Military Trauma Management Training: Protocol for a Systematic Review

**DOI:** 10.2196/45969

**Published:** 2023-09-08

**Authors:** Natalia Stathakarou, Andrzej A Kononowicz, Cara Swain, Klas Karlgren

**Affiliations:** 1 Learning, Informatics, Management and Ethics Karolinska Institutet Stockholm Sweden; 2 Department of Bioinformatics and Telemedicine Jagiellonian University Medical College Kraków Poland; 3 Academic Department of Military Surgery and Trauma Royal Centre for Defence Medicine Birmingham United Kingdom; 4 Institute of Naval Medicine Alverstoke, Gosport United Kingdom; 5 Department of Research, Education and Development and Innovation Södersjukhuset Stockholm Sweden; 6 Faculty of Health and Social Sciences Department of Health and Functioning Western Norway University of Applied Sciences Bergen Norway

**Keywords:** gamification, game elements, military medicine, trauma, medical education, military training, systematic review, game, gaming, simulation

## Abstract

**Background:**

Military trauma teams are commonly operating in civilian hospitals during peacetime; in a war situation they must adjust their practices to the austere conditions. Simulations can replicate austere conditions to allow training in a safe environment that tolerates errors. Gamification, understood as the use of game elements to motivate and engage learners in nongame contexts, is gaining interest in medical education and military training. Applying game elements in the design of military trauma management simulations has the potential to provide learners with active learning opportunities and prepare them for providing medical services under austere conditions. Although gamification is known for its engaging and motivational benefits, there are controversies about its pedagogical value. The controversies can be attributed to the fact that various gamification strategies may consist of a different combination of game elements, leading to different outcomes.

**Objective:**

This systematic review aims to understand how game elements are used in the design of simulations in military trauma management training and their reported outcomes.

**Methods:**

We have designed a search strategy for the purpose of the review. Two researchers will independently assess the identified studies based on the defined inclusion and exclusion criteria. The selection process will be represented using a PRISMA (Preferred Reporting Items for Systematic Reviews and Meta-Analyses) flow diagram. The search will be repeated and updated as necessary prior to publication of the review. Two reviewers will independently extract and manage the data for each of the articles using a structured data extraction form. Any disagreement that arises between reviewers will be resolved through discussion, and a third review author will be consulted when needed. We are going to conduct a thematic synthesis of the extracted game element descriptions. The results are going to be presented in a diagrammatic or tabular form, alongside a narrative summary. The quality of the studies will be assessed.

**Results:**

We implemented and tested the developed search strategy in May 2023. We retrieved 1168 study abstracts, which were reduced to 630 abstracts after deduplication. We have piloted the screening on 20% (126/630) of the identified abstracts in groups of 2 reviewers.

**Conclusions:**

Although gamification has the potential to motivate learners in various ways, there is a lack of understanding about specific game elements and how they can inform instructional design in different contexts. Our findings will increase the understanding of how game elements are used in the design of simulations in military trauma management training and, thus, contribute to more effective development of future simulations.

**International Registered Report Identifier (IRRID):**

DERR1-10.2196/45969

## Introduction

### Background

Trauma injury is one of the leading causes of death in Western countries and is a significant threat to public health [1]. Hemorrhage is the leading cause of preventable death in both military and civilian trauma [2]. In many nations, including Sweden, trauma team members work in both civilian and military settings, with military trauma teams commonly operating in civilian hospitals during peacetime [3]. Usually, the multidisciplinary trauma team consists of a variety of specialized health care professionals who collaborate with first responders and medics. Trauma patient outcomes are highly impacted by the performance of trauma teams, requiring a combination of technical and nontechnical skills. In a war situation, the trauma teams must adjust their practices to the austere conditions outside the fully equipped civilian hospital, operate with reduced numbers of team members, and deal with significantly higher numbers of patients than they might be accustomed to.

Prehospital trauma care differs significantly between a civilian and a deployed setting [4]. In the latter, resources in terms of personnel and equipment are limited, and there is a risk that multiple injured casualties can overburden the capacity of the resources. In the deployed setting the environment may hinder the provision of trauma management, due to challenging weather conditions and darkness. The environment may be hostile with the risk of gunfire hindering immediate patient care [5,6]. Additional tactical considerations may limit the possibilities for providing equipment and resources, as well as evacuating the patients requiring further care.

According to a recently published study [6], research and education may overlook the conditions of a truly austere environment. Health care professionals should be adequately trained to use a wide range of skills in environments with constrained resources in contemporary conflicts. Training does not stop at medical personnel but extends to the soldiers who are often the first responders. All combatants should be familiar with tourniquets and basic life support techniques. However, the learning opportunities to practice such skills are limited, due to the lack of exposure to trauma patients in austere conditions. In most cases, military medical personnel receive training in civilian settings for the tasks they will face in conflict. Simulation training has the potential to support the training and amend this imbalance [3].

Simulation can replicate austere conditions and allow the training in a safe environment that tolerates errors [7]. Simulation may refer to various technologies, from virtual reality equipment to mannequins and field exercises. Some simulation types can be resource-intensive regarding cost, facilities, faculty time, and the alignment of curricular and learners’ schedules while others can provide more flexibility and scalability [8]. Different simulation technologies can be used to train different competencies in trauma [9]. A previous study [10] identified examples of emerging technologies used for trauma training that have the potential to support the education and training toward challenges that civilian trauma teams face when moving to an austere environment. Examples included virtual reality, virtual reality combined with haptics and manikins or requiring special equipment, virtual patients, and gamification.

Gamification is gaining interest in medical education [11] and military training [8,12]. Gamification is a strategy in simulation design understood as “the use of game mechanics as a further dimension... in an endeavor to nudge participants to perform certain actions, by adopting a playful attitude” [13]. For instance, the learner might be required to solve a problem under time pressure, compete or collaborate with other users, and earn points or badges [14].

Incorporating game elements in health professions education can increase motivation and improve learning outcomes, specifically when employing game elements that promote learning behaviors and attitudes [15]. Applying game elements in the design of military trauma management simulation has the potential to provide learners with active learning opportunities to prepare for providing medical services under austere conditions [12].

Previous work analyzing game elements in health care education, as well as in education in general, has led to frameworks that can support the design and evaluation of gamification design in learning environments [14,16]. Although gamification is known for its engaging and motivational benefits, there are controversies about its pedagogical value [13,15]. The controversies can be attributed to the fact that various gamification strategies may consist of a different combination of game elements, leading to different outcomes [17]. Although gamification has the potential to motivate learners in various ways there is a lack of understanding about specific game elements and how they can inform the instructional design in different contexts [17].

### Aim

This systematic review aims to understand how game elements are used in the design of simulations in military trauma management training and their reported outcomes. Our research questions are:

1. What game elements are used in the design of educational simulations in the context of military trauma management?
2. How are the identified game elements used?
3. What is the purpose of using game elements in the designing of educational simulations in military trauma management?
4. What outcomes are reported related to the game elements?

## Methods

### Inclusion and Exclusion Criteria

We will include both qualitative and quantitative empirical and design studies that address different types of simulation interventions, identified in our previous study [10], which incorporate game elements. Game elements will be identified using published gamification frameworks [14,16]. We will include game elements that support the education and training of military trauma management. The simulation interventions of the studies included should have an educational purpose in the context of military trauma management.

Studies in which participants are passively observing rather than actively interacting with the intervention will be excluded. Studies that are using interventions primarily for patient education, rehabilitation, telementoring, treatment, surgical assistance, or planning are not within the scope of this review. We will exclude studies where the training only focuses on individual body parts. We will exclude studies that gamification is only used for assessment unless the assessment has an educational purpose and is not intended to assess the outcome of the study. Table 1 outlines our inclusion and exclusion criteria.

**Table 1:** Inclusion and exclusion criteria.

Criteria	Include	Exclude
Context and participants	All participants that are receiving training in the context of military trauma management. Participants are military medicine service providers (physicians, nurses, health care–qualified soldiers) at all stages of education. Included are also military personnel without health care qualifications who are trained to perform lifesaving procedures under combat conditions. Included are also health care personnel receiving military medicine education which requires immediate response and collaboration under austere conditions.	Studies involving only civilian health care professionals without a clear link to military—for example, residents in an emergency medicine program participating in a gamified trauma simulation.
Education and training	All forms of learning opportunities both implemented in formal curricula and one-time educational events with the intention to gain knowledge, skills or improve attitudes.	Scenarios where the gamified simulation is used merely as a form of outcome measurement and the educational impact of the assessment itself is not considered. Simulations containing game elements with planned therapeutic health effect—for example, in treatment of posttraumatic stress disorder; those that aim to be used for diagnostic purposes or as motivational elements to improve compliance with treatment regimen. Excluded are also studies that are using interventions primarily for patient education, rehabilitation, telementoring, treatment, surgical assistance, or planning.
Trauma	Scenarios in which people were physically injured and require stabilizing, time-critical decisions with incomplete information, performance of concurrent medical aid tasks, multidisciplinary management, and collaboration. Both simple (one body system) and complex injuries (more than one body system) are included.	We exclude psychological trauma without physical injuries that required immediate attention. We also exclude studies that focus only on individual injured body parts without considering the context of the injury.
Simulation	Empirical or design studies including a simulation modality such as virtual and mixed reality, virtual worlds, digital and non-digital games, interactive scenarios such as virtual patients, mannequins, part task trainers, artificial wounds and cut suits, standardized patients, and deliberately designed realistic field exercises employing role-playing.	Studies where participants are passive observers without interacting in the simulation.
Game element	At least one game element is included in the simulation as defined by Maheu-Cadotte et al [14]. We will also include studies with game elements going beyond the classification framework by Maheu-Cadotte et al [14] provided the activity or feature is explicitly and detailed enough described as designed to introduce game elements into the simulation. The included simulations must have an interactive plot as a game element.	Simulations, where the plot is in the form of a case vignette without interactive unfolding during the intervention, are going to be excluded.

### Search Methods for Identification of Studies

We will explore the databases or search engines Medline (Ovid), PubMed, IEEE Xplore, ERIC, Web of Science, and ACM Digital Library CINAHL with the help of librarians at Karolinska Institutet. We will include all articles regardless of publication language.

### Searching Other Sources

For all included studies, we will search reference lists and conduct author and citation searches. We will search the lists of references of other systematic reviews that are identiﬁed while running our electronic searches.

### Selection of Studies

We will import all references identified to the Rayyan open-source web system. Two researchers will independently assess the identified studies based on the inclusion and exclusion criteria. Any disagreements will be resolved through discussion between the 2 reviewers. If no agreement can be reached, a third author will be consulted. The selection process will be represented using a PRISMA (Preferred Reporting Items for Systematic Reviews and Meta-Analyses) flow diagram, including the actual number and the inclusion and exclusion criteria of each stage of screening [18]. The search will be repeated and updated as necessary prior to publication of the review.

### Data Extraction and Management

Two reviewers will independently extract and manage the data for each of the articles using a structured data extraction form. The data extraction process will be piloted among the researchers. Any disagreement that arises between reviewers will be resolved through discussion and if needed mediation of a third review author. If any articles are in a language the authors are not familiar with, the text in a non-English language will be first automatically translated by an artificial intelligence–powered translation tool (DeepL; DeepL SE), and if needed, a researcher native to the language will be consulted. We deem this strategy to be sufficient as we aim to analyze the manifest presentation of game elements and are not investigating the latent, culture-based, meaning of game elements. The data extraction form will be based on our previous systematic review [19] and will include extraction categories such as research aim, learning objectives, setting, subject, group of learners, instruments used for assessment if any, outcome of the intervention, and will be extended by fields such as type of simulation, game elements, type of trauma, and austere factors. To answer the second and third research questions, qualitative data (ie, text passages describing the game element design and purpose and images like screenshots showing game elements) will be extracted for thematic synthesis. To provide answers to the first and fourth research questions, we will extract data based on prespecified categories, inspired by the identified gamification frameworks and former systematic reviews [14,16,19]; the data extraction form corresponding to these topics is enclosed in the Multimedia Appendix 1.

### Data Analysis, Synthesis, and Reporting

A qualitative data analysis combined with a thematic data synthesis will be conducted. In particular, the extracted data will be synthesized following the processes outlined by Thomas and Harden [20]. First, the data are going to be coded in a reductant way to encapsulate the meaning of the extracted text. Codes will be developed from the text and extend and modify prespecified codes adopted from the identified gamification frameworks [14,16]. Next, descriptive themes are going to be established to unify codes that have been identified in more than one study. In the third and last stage, analytical themes will be generated; this step will aim to interpret the identified descriptive themes in a way that can support answering of the research questions. The data synthesis will be performed by the first author (NS) and will be discussed and reviewed by all authors. The results are going to be presented in a diagrammatic or tabular form, alongside a narrative summary.

### Quality Appraisal

The quality of studies will be assessed with the use of the Medical Education Research Study Quality Instrument (MERSQI) [21] and the grid suggested by Côté and Turgeon [22].

## Results

We implemented and tested the developed search strategy in May 2023. We retrieved 1168 study abstracts, which were reduced to 630 abstracts after deduplication. We have piloted the screening on 20% (n=126) of identified abstracts in groups of 2 reviewers. Our search strategy may be found in Multimedia Appendix 2.

## Discussion

### Anticipated Findings

Gamification has the potential to motivate learners in various ways, and it is showing promising results in improving forward combat casualty care performance in a simulated tactical environment [23,24]. The effectiveness of games as instructional tools has been debated over the years as there is a lack of understanding about specific game elements and how they can influence the reported outcomes. A recent systematic literature review about the application of virtual reality and haptic interfaces for civilian and military open trauma surgery training reports mixed and poor-quality evidence about their realization in education [25]; we envision that an enhanced understanding of game elements in educational simulations would inform the design of educational technologies and simulations.

Our starting point to interpret and configure the game elements is based on existing frameworks to understand gamification as a concept [14,16]. Several definitions exist for gamification, with the most widespread definition to be from Deterding et al [26] as the use of game elements in nongame contexts. In this protocol, we use a relatively new definition proposed by Pfeiffer et al [13] that emphasizes the principle of nudging [27] the participants with playful elements to engage in simulation, and therefore it can be understood as a tool for triggering positive behaviors towards learning.

We acknowledge that our backgrounds may influence the data analysis and synthesis. NS and AAK have a background in health informatics and research interests in educational technologies, specifically virtual patients; CS is a military surgeon and researcher in surgical education; and KK has a background in interaction design, technology-enhanced learning, and medical education.

### Limitations

A potential limitation of the upcoming systematic review might be that evidence published in gray literature is not going to be retrieved; we might therefore overlook evidence about gamification applications that have not been reported in peer-reviewed journals. Additionally, we may not retrieve papers that did not include keywords that we have a priori associated with game elements. To control this challenge, we have collaborated with a team of professional librarians to design a complex search strategy and will hand-search references in included studies.

### Conclusions

To our knowledge, this is going to be the first systematic review to explore game elements in the design of educational simulations in the context of military trauma management. Our findings will increase the understanding of how game elements are used in the design of simulations in military trauma management training and by that hopefully contribute to more effective development of future simulations.

## Appendix

Multimedia Appendix 1 Data extraction sheet and initial coding frame for game elements and outcomes in the included studies.

Multimedia Appendix 2 Search strategy and number of results retrieved.

## Abbreviations

MERSQI: Medical Education Research Study Quality Instrument
PRISMA: Preferred Reporting Items for Systematic Reviews and Meta-Analyses

## Data Availability

The data sets generated or analyzed during this study are available from the corresponding author on reasonable request.
