# Supplementary material for: Game Elements in the Design of Simulations in Military Trauma Management Training: Protocol for a Systematic Review
Source: JMIR Res Protoc. 2023 Sep 8;12:e45969. doi: 10.2196/45969 (PMC10517381; doi:10.2196/45969)
Supplement: Multimedia Appendix 1 [file resprot_v12i1e45969_app1.docx]

Multimedia Appendix 1. Data extraction sheet and initial coding frame for game elements and outcomes in the included studies.

| Data field | | Data type |
| --- | --- | --- |
| Learning theory about the use of gamification or game elements if any | | Free text |
| **Presence of following game elements: (mark the relevant description in the included paper)** | | |
|  | Avatars | Yes / No / Unclear |
|  | Badges for achievement | Yes / No / Unclear |
|  | Content unlocking | Yes / No / Unclear |
|  | Difficulty adaption | Yes / No / Unclear |
|  | Hints | Yes / No / Unclear |
|  | Leaderboard | Yes / No / Unclear |
|  | Performance tables or graphs | Yes / No / Unclear |
|  | Plot | Yes / No / Unclear |
|  | Points | Yes / No / Unclear |
|  | Teams | Yes / No / Unclear |
|  | Time pressure | Yes / No / Unclear |
|  | Virtual goods | Yes / No / Unclear |
|  | Description of other game elements | Free text |
| Please describe how the game elements marked above are used in the educational intervention | | Free text |
| Was the gamification used to support the immersion of austere factors? | | Yes / No / Unclear |
| If yes above, describe how? | | Free text |
| What is the purpose of using gamification elements in the designing of educational simulations in military trauma management? | | Free text |
| **If multiple outcome complete for each domain separately:** | | |
|  | Domain of learning | Knowledge / Skills / Attitudes / Satisfaction / Economic analysis / Patient outcome / Combined / Other / Unclear |
|  | Specific type of outcome reported | Basic Knowledge / Clinical reasoning / Team training / Procedural & Basic skills / Patient communication / Other / Unclear / Not applicable |
| If other or combined, specify | | Free text |
| Findings reported by the authors | | Free text |
| Other comments | | Free text |

References:

[1] Maheu-Cadotte MA, Cossette S, Dubé V, Fontaine G, Mailhot T, Lavoie P, et al. Effectiveness of serious games and impact of design elements on engagement and educational outcomes in healthcare professionals and students: A systematic review and meta-Analysis protocol. Vol. 8, BMJ Open. 2018.

[2] Toda AM, Klock ACT, Oliveira W, Palomino PT, Rodrigues L, Shi L, et al. Analysing gamification elements in educational environments using an existing Gamification taxonomy. Smart Learning Environments. 2019; 6(1).

[3] Kononowicz AA, Woodham LA, Edelbring S, Stathakarou N, Davies D, Saxena N, et al. Virtual patient simulations in health professions education: Systematic review and meta-analysis by the digital health education collaboration. Journal of Medical Internet Research. 2019.
