# Supplementary material for: Game Elements in the Design of Simulations in Military Trauma Management Training: Protocol for a Systematic Review
Source: JMIR Res Protoc. 2023 Sep 8;12:e45969. doi: 10.2196/45969 (PMC10517381; doi:10.2196/45969)
Supplement: Multimedia Appendix 2 [file resprot_v12i1e45969_app2.docx]

Multimedia Appendix 2. Search strategy and number of results retrieved.

Date: May, 2023

Databases:

1. Medline (Ovid)
2. IEEE Xplore
3. ERIC
4. Web of Science
5. ACM Digital Library
6. CINAHL
7. PubMed

Total number of hits:

- Before deduplication: 1,168
- After deduplication: 630

Comments:

Deduplication based on the method described in:
Bramer, W. M., Giustini, D., de Jonge, G. B., Holland, L., & Bekhuis, T. (2016). De-duplication of database search results for systematic reviews in EndNote. *Journal of the Medical Library Association: JMLA*, 104(3), 240–243. doi:10.3163/1536-5050.104.3.014

One final, extra step was added to compare DOIs.

1. Medline

| Interface: Ovid MEDLINE(R) ALL  Date of Search: May 23, 2023  Number of hits: 391  Comment: In Ovid, two or more words are automatically searched as phrases; i.e. no quotation marks are needed | Field labels   - exp/ = exploded MeSH term - / = nonexploded MeSH term - .ti,ab,kf. = title, abstract, and author keywords - adjx = within x words, regardless of order - * = truncation of word for alternate endings |
| --- | --- |
| Database(s): **Ovid MEDLINE(R) ALL**1946 to May 22, 2023 Search Strategy:   \| **#** \| **Searches** \| **Results** \| \| --- \| --- \| --- \| \| 1 \| Computer Simulation/ \| 210031 \| \| 2 \| exp Augmented Reality/ \| 1134 \| \| 3 \| exp Virtual Reality/ \| 5574 \| \| 4 \| exp Ambient Intelligence/ \| 50 \| \| 5 \| exp Computer-Assisted Instruction/ \| 12542 \| \| 6 \| exp Video Games/ \| 7236 \| \| 7 \| exp Simulation Training/ \| 11575 \| \| 8 \| (gamification* or game* or gaming or virtual reality or virtual patient* or virtual environment or virtual patient* or augmented reality or augmented virtuality or mixed reality or merged reality or full immersion).ab,kf,ti. \| 99945 \| \| 9 \| 1 or 2 or 3 or 4 or 5 or 6 or 7 or 8 \| 327379 \| \| 10 \| exp Emergency Medicine/ or exp Disaster Medicine/ \| 16419 \| \| 11 \| exp Emergency Responders/ \| 15372 \| \| 12 \| exp Emergency Nursing/ \| 7411 \| \| 13 \| exp Emergency Treatment/ \| 134750 \| \| 14 \| exp Trauma Centers/ \| 13083 \| \| 15 \| exp "Wounds and Injuries"/ \| 1005366 \| \| 16 \| (trauma care or trauma management or bleeding or gun shot* or gunshot* or blast injur* or wound* or surgery or CPR or resuscitation or first aid or first responder or paramedic* or accident* or emergency medicine).ab,kf,ti. \| 2098465 \| \| 17 \| 10 or 11 or 12 or 13 or 14 or 15 or 16 \| 2979973 \| \| 18 \| exp Military Medicine/ \| 30130 \| \| 19 \| exp Military Nursing/ \| 2284 \| \| 20 \| (defence medicine* or defense medicine* or combat or war or tactical or military).ti,ab,kf. \| 150038 \| \| 21 \| 18 or 19 or 20 \| 164432 \| \| 22 \| 9 and 17 and 21 \| 391 \| | |

2. IEEE Xplore

| Interface: embase.com  Date of Search: May 23, 2023  Number of hits: 67 | Field labels: All Metadata includes the abstract, index terms, and bibliographic citation data (such as document title, publication title, author, etc.). |
| --- | --- |
| ("All Metadata":gamification* OR "All Metadata":game* OR "All Metadata":gaming OR "All Metadata":"virtual reality" OR "All Metadata":"virtual patient*" OR "All Metadata":"virtual environment" OR "All Metadata":"virtual patient*" OR "All Metadata":"augmented reality" OR "All Metadata":"augmented virtuality" OR "All Metadata":"mixed reality" OR "All Metadata":"merged reality" OR "All Metadata":"full immersion")  AND  ("All Metadata":"trauma care" OR "All Metadata":"trauma management" OR "All Metadata":bleeding OR "All Metadata":"gun shot*" OR "All Metadata":gunshot* OR "All Metadata":"blast injur*" OR "All Metadata":wound* OR "All Metadata":surgery OR "All Metadata":CPR OR "All Metadata":resuscitation OR "All Metadata":"first aid" OR "All Metadata":"first responder" OR "All Metadata":paramedic* OR "All Metadata":accident* OR "All Metadata":"emergency medicine")  AND  ("All Metadata":"defence medicine*" OR "All Metadata":"defense medicine*" OR "All Metadata":combat OR "All Metadata":war OR "All Metadata":tactical OR "All Metadata":military) | |

3. ERIC

| Interface: ProQuest  Date of Search: May 23, 2023  Number of hits: 7 | Field labels: Search performed in all fields |
| --- | --- |
| (gamification* or game* or gaming or "virtual reality" or "virtual patient*" or "virtual environment" or "virtual patient*" or "augmented reality" or "augmented virtuality" or "mixed reality" or "merged reality" or "full immersion")  AND  ("trauma care" or "trauma management" or bleeding or "gun shot*" or gunshot* or "blast injur*" or wound* or surgery or CPR or resuscitation or "first aid" or "first responder" or paramedic* or accident* or "emergency medicine")  AND  ("defence medicine*" or "defense medicine*" or combat or war or tactical or military) | |

4. Web of Science Core Collection

| Interface: Clarivate Analytics  Date of Search: May 23, 2023  Number of hits: 164 | Field labels   - TS/Topic = title, abstract, author keywords, and Keywords Plus - NEAR/x = within x words, regardless of order - * = truncation of word for alternate endings   Note: sometimes “quotation marks” are needed for single search terms to avoid automatic term mapping (lemmatization). |
| --- | --- |
| gamification* or game* or gaming or "virtual reality" or "virtual patient*" or "virtual environment" or "virtual patient*" or "augmented reality" or "augmented virtuality" or "mixed reality" or "merged reality" or "full immersion" (Topic)  AND  "trauma care" or "trauma management" or bleeding or "gun shot*" or gunshot* or "blast injur*" or wound* or surgery or CPR or resuscitation or "first aid" or "first responder" or paramedic* or accident* or "emergency medicine" (Topic)  AND  "defence medicine*" or "defense medicine*" or combat or war or tactical or military (Topic) | |

5. ACM Digital Library

| Interface: ACM Digital Library  Date of Search: May 23, 2023  Number of hits: 11 | Notes: You need to preform searches for different fields sequentially |
| --- | --- |
| [[Title: gamification*] OR [Title: game*] OR [Title: gaming] OR [Title: "virtual reality"] OR [Title: "virtual patient*"] OR [Title: "virtual environment"] OR [Title: "virtual patient*"] OR [Title: "augmented reality"] OR [Title: "augmented virtuality"] OR [Title: "mixed reality"] OR [Title: "merged reality"] OR [Title: "full immersion"]] AND [[Title: "trauma care"] OR [Title: "trauma management"] OR [Title: bleeding] OR [Title: "gun shot*"] OR [Title: gunshot*] OR [Title: "blast injur*"] OR [Title: wound*] OR [Title: surgery] OR [Title: cpr] OR [Title: resuscitation] OR [Title: "first aid"] OR [Title: "first responder"] OR [Title: paramedic*] OR [Title: accident*] OR [Title: "emergency medicine"]] AND [[Title: "defence medicine*"] OR [Title: "defense medicine*"] OR [Title: combat] OR [Title: war] OR [Title: tactical] OR [Title: military]]  0 st  [[Abstract: gamification*] OR [Abstract: game*] OR [Abstract: gaming] OR [Abstract: "virtual reality"] OR [Abstract: "virtual patient*"] OR [Abstract: "virtual environment"] OR [Abstract: "virtual patient*"] OR [Abstract: "augmented reality"] OR [Abstract: "augmented virtuality"] OR [Abstract: "mixed reality"] OR [Abstract: "merged reality"] OR [Abstract: "full immersion"]] AND [[Abstract: "trauma care"] OR [Abstract: "trauma management"] OR [Abstract: bleeding] OR [Abstract: "gun shot*"] OR [Abstract: gunshot*] OR [Abstract: "blast injur*"] OR [Abstract: wound*] OR [Abstract: surgery] OR [Abstract: cpr] OR [Abstract: resuscitation] OR [Abstract: "first aid"] OR [Abstract: "first responder"] OR [Abstract: paramedic*] OR [Abstract: accident*] OR [Abstract: "emergency medicine"]] AND [[Abstract: "defence medicine*"] OR [Abstract: "defense medicine*"] OR [Abstract: combat] OR [Abstract: war] OR [Abstract: tactical] OR [Abstract: military]]  11 st | |

6. Cinahl

| Interface: Ebsco  Date of Search: May 23, 2023  Number of hits: 145 | Field labels   - MH+ = exploded Cinahl Heading - MH = nonexploded Cinahl Heading - TI = title - AB = abstract - Nx = within x words, regardless of order - * = truncation of word for alternate endings |
| --- | --- |
| \| # \| Query \| Results \| \| --- \| --- \| --- \| \| S25 \| S9 AND S18 AND S24 \| 145 \| \| S24 \| S19 OR S20 OR S23 \| 51,348 \| \| S23 \| S21 OR S22 \| 47,279 \| \| S22 \| AB (defence medicine* or defense medicine* or combat or war or tactical or military) \| 36,233 \| \| S21 \| TI (defence medicine* or defense medicine* or combat or war or tactical or military) \| 21,157 \| \| S20 \| (MH "Military Nursing") \| 3,406 \| \| S19 \| (MH "Military Medicine") \| 4,863 \| \| S18 \| S10 OR S11 OR S12 OR S13 OR S14 OR S17 \| 815,771 \| \| S17 \| S15 OR S16 \| 497,911 \| \| S16 \| AB (trauma care or trauma management or bleeding or gun shot* or gunshot* or blast injur* or wound* or surgery or CPR or resuscitation or first aid or first responder or paramedic* or accident* or emergency medicine or emergency responders or disaster medicine) \| 403,279 \| \| S15 \| TI (trauma care or trauma management or bleeding or gun shot* or gunshot* or blast injur* or wound* or surgery or CPR or resuscitation or first aid or first responder or paramedic* or accident* or emergency medicine or emergency responders or disaster medicine) \| 186,937 \| \| S14 \| (MH "Wounds and Injuries+") \| 327,293 \| \| S13 \| (MH "Trauma Centers") \| 7,694 \| \| S12 \| (MH "Emergency Treatment+") \| 64,593 \| \| S11 \| (MH "Emergency Nursing+") \| 15,374 \| \| S10 \| (MH "Emergency Medicine") \| 13,243 \| \| S9 \| S1 OR S2 OR S3 OR S4 OR S5 OR S8 \| 71,914 \| \| S8 \| S6 OR S7 \| 42,043 \| \| S7 \| AB ("ambient intelligence" or gamification* or game* or gaming or virtual reality or virtual patient* or virtual environment or virtual patient* or augmented reality or augmented virtuality or mixed reality or merged reality or full immersion or "simulation training") \| 31,755 \| \| S6 \| TI ("ambient intelligence" or gamification* or game* or gaming or virtual reality or virtual patient* or virtual environment or virtual patient* or augmented reality or augmented virtuality or mixed reality or merged reality or full immersion or "simulation training") \| 19,544 \| \| S5 \| (MH "Video Games+") \| 5,764 \| \| S4 \| (MH "Computer Assisted Instruction") \| 8,129 \| \| S3 \| (MH "Virtual Reality+") \| 7,594 \| \| S2 \| (MH "Augmented Reality") \| 463 \| \| S1 \| (MH "Computer Simulation") \| 19,316 \| | |

7. PubMed

| Interface: PubMed  Date of Search: May 23, 2023  Number of hits: 383 | Field labels   - Mesh = exploded MeSH Term - Mesh:NoExp = nonexploded MeSH Term - tiab= title/abstract - * = truncation of word for alternate endings |
| --- | --- |
| \|  \| **Query** \| **Results** \| \| --- \| --- \| --- \| \| 1 \| "Computer Simulation"[Mesh:NoExp] \| 209,833 \| \| 2 \| "Augmented Reality"[Mesh] \| 1,131 \| \| 3 \| "Virtual Reality"[Mesh] \| 5,557 \| \| 4 \| "Ambient Intelligence"[Mesh] \| 50 \| \| 5 \| "Computer-Assisted Instruction"[Mesh] \| 12,540 \| \| 6 \| "Video Games"[Mesh] \| 7,234 \| \| 7 \| "Simulation Training"[Mesh] \| 11,572 \| \| 8 \| (gamification*[tiab] OR game*[tiab] OR gaming[tiab] OR "virtual reality"[tiab] OR "virtual patient*"[tiab] OR "virtual environment"[tiab] OR "virtual patient*"[tiab] OR "augmented reality"[tiab] OR "augmented virtuality"[tiab] OR "mixed reality"[tiab] OR "merged reality"[tiab] OR "full immersion"[tiab]) \| 100,110 \| \| 9 \| #1 OR #2 OR #3 OR #4 OR #5 OR #6 OR #7 OR #8 \| 327,333 \| \| 10 \| "Emergency Medicine"[Mesh] OR "Disaster Medicine"[Mesh] \| 16,414 \| \| 11 \| "Emergency Responders"[Mesh] \| 15,350 \| \| 12 \| "Emergency Nursing"[Mesh] \| 7,410 \| \| 13 \| "Emergency Treatment"[Mesh] \| 134,673 \| \| 14 \| "Trauma Centers"[Mesh] \| 13,069 \| \| 15 \| "Wounds and Injuries"[Mesh] \| 1,004,924 \| \| 16 \| ("trauma care"[tiab] OR "trauma management"[tiab] OR bleeding[tiab] OR "gun shot*"[tiab] OR gunshot*[tiab] OR "blast injur*"[tiab] OR wound*[tiab] OR surgery[tiab] OR CPR[tiab] OR resuscitation[tiab] OR "first aid"[tiab] OR "first responder"[tiab] OR paramedic*[tiab] OR accident*[tiab] OR "emergency medicine"[tiab]) \| 2,098,721 \| \| 17 \| #10 OR #11 OR #12 OR #13 OR #14 OR #15 OR #16 \| 2,979,729 \| \| 18 \| "Military Medicine"[Mesh] \| 30,126 \| \| 19 \| "Military Nursing"[Mesh] \| 2,284 \| \| 20 \| ("defence medicine*"[tiab] OR "defense medicine*"[tiab] OR combat[tiab] OR war[tiab] OR tactical[tiab] OR military[tiab]) \| 144,778 \| \| 21 \| #18 OR #19 OR #20 \| 159,149 \| \| 22 \| #9 AND #17 AND #21 \| 383 \| | |
